# Motion corrected sensitivity encoded isotropic projection reconstruction (SNIPR) for whole-heart coronary MRA

**DOI:** 10.1186/1532-429X-15-S1-E65

**Published:** 2013-01-30

**Authors:** Jianing Pang, Behzad Sharif, Reza Arsanjani, Louise E Thomson, John D Friedman, Daniel S Berman, Debiao Li

**Affiliations:** 1 Biomedical Imaging Research Institute, Cedars Sinai Medical Center, Los Angeles, CA, USA; 2 Radiology and Biomedical Engineering, Northwestern University, Chicago, IL, USA; 3 Bioengineering, University of California, Los Angeles, CA, USA

## Background

As has been shown recently, the use of undersampled 3D projection reconstruction (3DPR) and image-based motion correction with 100% acquisition efficiency [1,2] enables highly accelerated coronary MRA (CMRA) with high isotropic resolution. However, streaking artifact from undersampling significantly reduces the apparent signal-to-noise ratio (SNR). Moreover, affine motion correction distorts the k-space trajectory, hence introducing more streaking. In this work, the SNIPR reconstruction [3], a self-calibrated sensitivity encoding scheme for 3DPR, is integrated with image-based motion correction to reduce streaking artifacts and thus improve image quality.

## Methods

MR data was collected using an ECG-triggered, T2-prepared, fat-saturated bSSFP pulse sequence with 3DPR trajectory and a 12 channel receiver coil array (TR/TE = 3.2 ms/1.6 ms, FOV=400 mm^3^, matrix size=384^3^, flip angle=90°, readout bandwidth=900 Hz/pixel). Three datasets were collected from each subject in random order with 5800, 11500 or 20500 lines (imaging times: 2.8±0.3, 5.6±0.6 and 9.5±1.2 min). Retrospective respiratory motion compensation was performed as previously described in [2]. The coil sensitivity maps were calculated with the motion corrected data using Walsh's method [4]. SNIPR extends the generalized SENSE framework described in [5] to 3D for iterative image reconstruction. Healthy volunteer scans (N=4) were performed successfully on a clinical 1.5T scanner (MAGNETOM Avanto, Siemens AG Healthcare, Erlangen, Germany) with IRB approval and written consent. Offline reconstruction was implemented in MATLAB (MathWorks, Natick, MA). Image quality scores were assigned by an experienced reader blinded to the underlying techniques.

## Results

**Figure 1:**
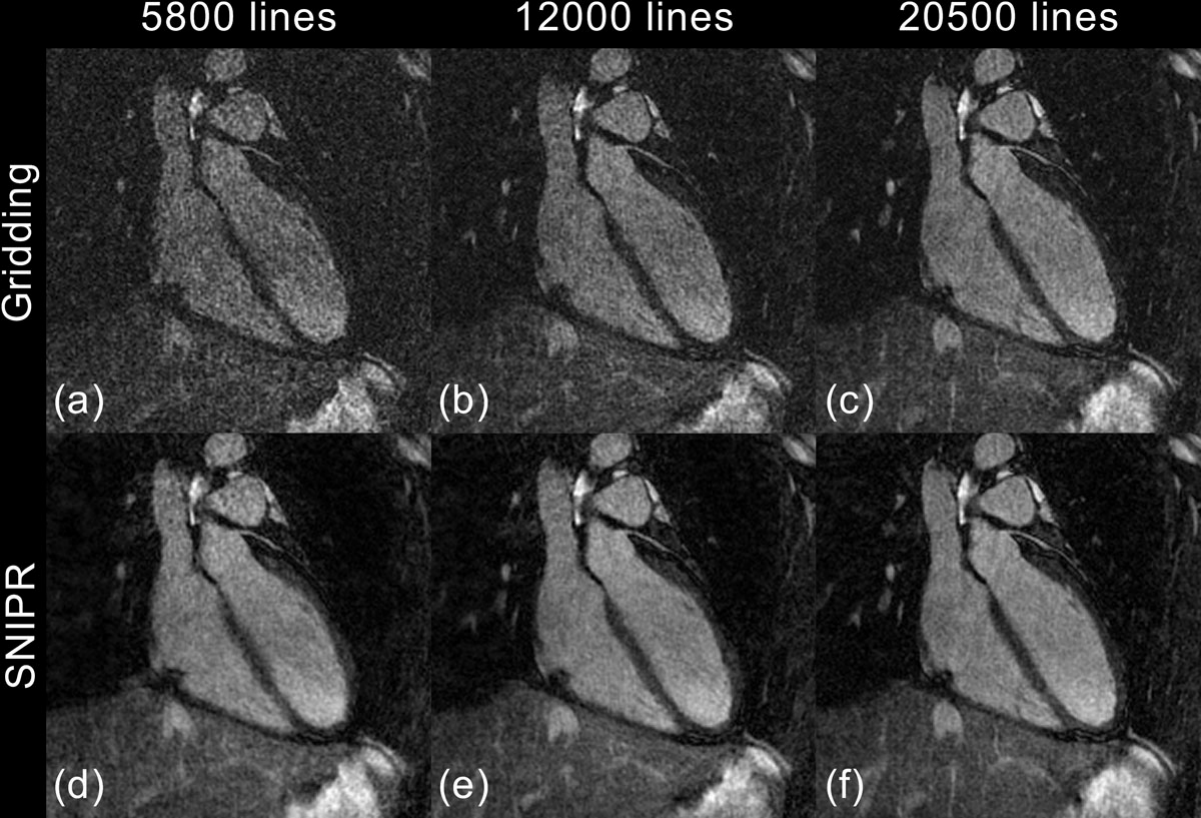
Comparing gridding and SNIPR. For various undersampling ratios, SNIPR significantly reduces streaking artifact, resulting in improved SNR.

The standard gridding shows significant streaking artifacts that increase with fewer lines, while SNIPR greatly reduces the amount of streaking in all datasets, improving the apparent SNR (Figure 1). Notably, the image qualities of 12000 and 20500 lines have no significant difference with SNIPR, although with 5800 lines there is a certain degree of blurring; this can be due to poor conditioning of the linear system, error in motion correction, and/or error in sensitivity maps. Table 1 shows the subjective image quality scores.

**Table 1:** Image quality score results (range: 0-4) for different reconstruction methods and undersampling factors.

	Gridding, 5800 lines	SNIPR, 5800 lines	Gridding, 12000 lines	SNIPR, 12000 lines	Gridding, 20500 lines	SNIPR, 20500 lines
Score	0.8±0.4	1.1±0.2	1.9±0.7	2.5±0.4	2.2±0.8	2.5±0.7

## Conclusions

We have described a self-calibrated, motion-corrected 3D non-Cartesian sensitivity encoding reconstruction that greatly reduces streaking artifacts from undersampled 3DPR acquisitions, with isotropic 1 mm^3^ resolution and a 6 minutes scan time for whole-heart CMRA. Further studies are needed to characterize the noise performance of the proposed method. Also, the final image quality will benefit from improvements in motion correction and sensitivity map estimation, especially for highly undersampled datasets.

## Funding

National Institute of Health grants nos. NIBIB EB002623 and NHLBI HL38698.AHA Postdoctoral Fellowship Award 11POST7390063.
